# Hox and Wnt pattern the primary body axis of an anthozoan cnidarian before gastrulation

**DOI:** 10.1038/s41467-018-04184-x

**Published:** 2018-05-22

**Authors:** Timothy Q. DuBuc, Thomas B. Stephenson, Amber Q. Rock, Mark Q. Martindale

**Affiliations:** 1 Centre for Chromosome Biology, Bioscience Building, National University of Ireland Galway, Galway, H91 RTR6 Ireland; 2 Whitney Lab for Marine Bioscience and the Department of Biology, University of Florida, St. Augustine, FL 32080 USA

## Abstract

*Hox* gene transcription factors are important regulators of positional identity along the anterior–posterior axis in bilaterian animals. Cnidarians (e.g., sea anemones, corals, and hydroids) are the sister group to the Bilateria and possess genes related to both anterior and central/posterior class *Hox* genes. Here we report a previously unrecognized domain of *Hox* expression in the starlet sea anemone, *Nematostella vectensis*, beginning at early blastula stages. We explore the relationship of two opposing *Hox* genes (*NvAx6*/*NvAx1*) expressed on each side of the blastula during early development. Functional perturbation reveals that *NvAx6* and *NvAx1* not only regulate their respective expression domains, but also interact with Wnt genes to pattern the entire oral–aboral axis. These findings suggest an ancient link between *Hox*/Wnt patterning during axis formation and indicate that oral–aboral domains are likely established during blastula formation in anthozoan cnidarians.

## Abstract (web summary)

*Hox* genes regulate anterior–posterior axis formation but their role in cnidarians is unclear. Here, the authors disrupt *Hox* genes *NvAx1* and *NvAx6* in the starlet sea anemone, *Nematostella vectensis*, showing antagonist function in patterning the oral–aboral axis and a link to Wnt signaling.

## Introduction

**Fig. 1:**
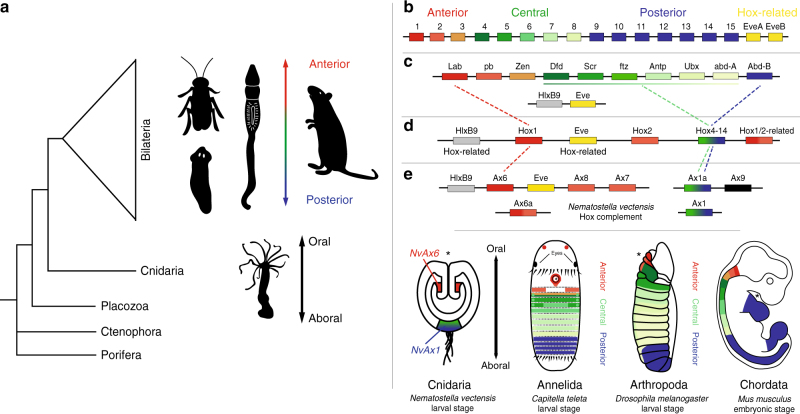
Anterior–posterior patterning and the emergence of a *Hox* cluster. **a** Bilaterians are classically defined by an anterior–posterior axis perpendicular to the dorsal ventral axis. Cnidarians are the sister taxa to bilaterians and are the only basal lineage to have a diverse cluster of *Hox* genes. **b** The common ancestor of the deuterostome lineage likely had a *Hox* cluster consisting of 14–15 *Hox* genes, closely associated with the homeobox gene *Eve*^18^. **c** Evidence from the protostome, *Tribolium castaneum*, suggests that the protostome ancestor also had an intact *Hox* cluster consisting of at least 10 linked *Hox* genes^17,70^. **d** The cnidarian ancestor had both anterior (*Hox*1 and *Hox*2) and central/posterior (*Hox*9–13) class *Hox* genes^22^. **e** The *Hox* complement of the anthozoan cnidarian, *Nematostella vectensis*, has phylogenetically anterior (*NvAx6*, *NvAx6a*, *NvAx7*, and *NvAx8*) and central/posterior (*NvAx1* and *NvAx1a*) *Hox* genes^14,15^. Depiction of *Hox* expression along the oral–aboral axis of a cnidarian, and the anterior–posterior axis of invertebrates and vertebrates. The anterior (*NvAx6*) and central/posterior (*NvAx1*) *Hox* genes of *Nematostella* are expressed along the oral–aboral axis during larval development. Regions of anterior, central, and posterior *Hox* expression are designated with shades of red, green, and blue, respectively. Asterisk indicates site of mouth formation

H*ox* genes are a specific family of homeobox-containing transcription factors that have been studied extensively in several clades of bilaterally symmetrical animals (“bilaterians”, Fig. 1a). Originally discovered in *Drosophila*^1^, *Hox* genes play an important role in establishing segment identity along the anterior–posterior (A–P) axis during development and are conserved in all bilaterian lineages including vertebrates^2^. *Hox* genes are often clustered together along contiguous stretches of an animal’s genome^3^, and are classified into three paralogy groups: anterior (*Hox*1–3), central (*Hox*4–8), and posterior (*Hox*9–13) (Fig. 1b). Early comparisons of vertebrate and insect *Hox* sequences revealed that homologous *Hox* genes occupy similar locations in the 3′–5′ topology of the cluster^4^ and gene expression analysis further revealed that a gene’s position in the cluster correlated with its expression profile along the A–P axis. Although there are derivations in the organization and distribution of *Hox* genes among bilaterians, *Hox* genes are consistently expressed in anterior-to-posterior territories as reflected by their phylogenic ancestry^5–11^. Furthermore, duplications (vertebrates) and fragmentation of *Hox* clusters (invertebrates) likely had a strong impact on the radiation of animals^8,12^, although the origin and function of this gene family within the first invertebrates is less clear^13–16^.

Phylogenetic reconstructions of the bilaterian *Hox* gene compliment indicate that the common ancestor of most extant animals had a *Hox* cluster containing anterior, central, and posterior *Hox* genes^17,18^ (Fig. 1b, c). Although clustering is not an essential component of *Hox* functionality, it is widely associated with diverse animal clades from cnidarians and throughout Bilateria. Cnidarians (e.g., corals, anemones, and hydroids) are the sister group to the Bilateria^19^ (Fig. 1a) and possess bona fide *Hox* genes related to anterior and central/posterior *Hox* genes^14,15,20–23^. The cnidarian common ancestor likely had a *Hox* cluster consisting of at least two types of *Hox* genes, related to anterior and central/posterior^21,22^ (Fig. 1d, Supplementary Fig. 1). Definitive central class genes have yet to be found in a cnidarian. They first appear in the Xenoacoelomorph bilaterian lineage^24^ and likely expanded in protostome/deuterostome lineages (Fig. 1b, c, Supplementary Fig. 1). Currently, it is uncertain whether central class *Hox* genes arose in the bilaterian lineage from an ancestral central/posterior *Hox* gene, or were lost in the cnidarian lineage. Bona fide *Hox* genes have yet to be found in the genomes of earlier branching animal clades^25–28^, although some evidence suggests gene loss may have impacted this gene family^16^ and a greater effort needs to be made to survey the diverse group of extant cnidarians. Efforts to functionally characterize the cnidarian *Hox* complement are crucial for determining the pre-bilaterian role of these important developmental regulators.

Functional studies in the fly^1^ and mouse^29^ first showed that *Hox* genes are causally involved in establishing adult body structures from the region in which they are expressed, thus controlling regional identity along the A–P axis. *Hox* genes also interact indirectly with one another in overlapping domains, with posterior *Hox* genes having functional “dominance” over more anterior genes; however, flies also exhibit examples of anterior dominance^30^. mRNA expression studies of *Hox* genes in multiple cnidarian species suggest a discrete role in late larval patterning, yet the developmental function remains untested^13,15,20,21,31,32^. The *Hox* cluster of the starlet sea anemone *Nematostella* consists of two neighboring non-Hox homeobox genes (*NvHlxB9* and *NvEve*), along with putative orthologs to the anterior *Hox* genes *Hox*1 (*NvAx6*) and *Hox*2 (*NvAx*7 and *NvAx8*)^14,15^. Four additional *Hox* genes exist in the genome, consisting of: two central/posterior genes (*NvAx1* and *NvAx1a*), a pseudogene (*NvAx9*), and an additional anterior-like gene of indeterminate orthology (*NvAx6a*) (Fig. 1e). The assemblage of *Hox* clusters from different cnidarian genomes indicate that anthozoan cnidarians had a relatively intact *Hox* cluster, which has been fragmented in different species (Supplementary Fig. 1). Corals arguably have the most complete cluster consisting of both anterior and central/posterior orthologs^22^ (Supplementary Fig. 1c). Representative anemone species (*Nematostella* and *Aiptasia*) appear to have diverged from a cluster seen in some corals with a duplicated *Hox2* ortholog (*Ax7* and *Ax8*) (Supplementary Fig. 1d–e). An ortholog of *NvAx1* is present in all cnidarian genomes, yet there is no evidence of it being linked to a cnidarian cluster, suggesting it diverged long ago. In the case of *Hydra*, a high rate of cluster fragmentation is correlated with an expansion of central/posterior genes and less conservation among anterior-like genes^14,15^ (Supplementary Fig. 1f). Upcoming genome projects of other Hydrozoan cnidarians, *Clytia hemisphaerica* and two *Hydractinia* species, may reveal more details about the distribution of these genes. All of these findings are based on the idea that the ancestral condition was a state of *Hox* clustering, which is generally assumed to be the representative condition among bilaterian clades. A lack of functional data only allows us to speculate on the impact of cluster fragmentation within Cnidaria, and additional work is needed to resolve the impact of the cnidarian *Hox* diversification.

Previous in situ hybridization studies conducted on larval and juvenile stages of *Nematostella* revealed that the majority of *NvHox* genes are expressed in a staggered domain along the primary (oral–aboral) axis^15^ with the anterior *Hox* gene (*NvAx6)* expressed in a region corresponding to the pharyngeal nerve ring at the oral pole, and the central/posterior *Hox* gene (*NvAx1*) expressed in the larval apical tuft at the aboral tip of the planula (Fig. 1e). The rest of the *N*. *vectensis Hox* genes are expressed later in development at planula stages asymmetrically along one side of the endoderm within the oral–aboral domain separating *NvAx6* and *NvAx1*^15,20^. These data along with other studies in medusazoan cnidarians that possess complex metagenic lifecycles have created uncertainty as to whether cnidarian *Hox* genes have any bilaterian-like attributes. Early work using the cnidarian model system, *Hydra*, found a role in adult oral–aboral patterning^31,33,34^, while more recent findings concluded that the axial patterning role of *Hox* genes arose after the cnidarian bilaterian split^13,21,32^. Here we begin to dissect for the first time the functional role and hierarchy of *Hox* genes during cnidarian embryonic development and generate a presumptive molecular network for oral–aboral patterning.

## Results

### *Hox* genes are expressed prior to germ layer segregation

**Fig. 2:**
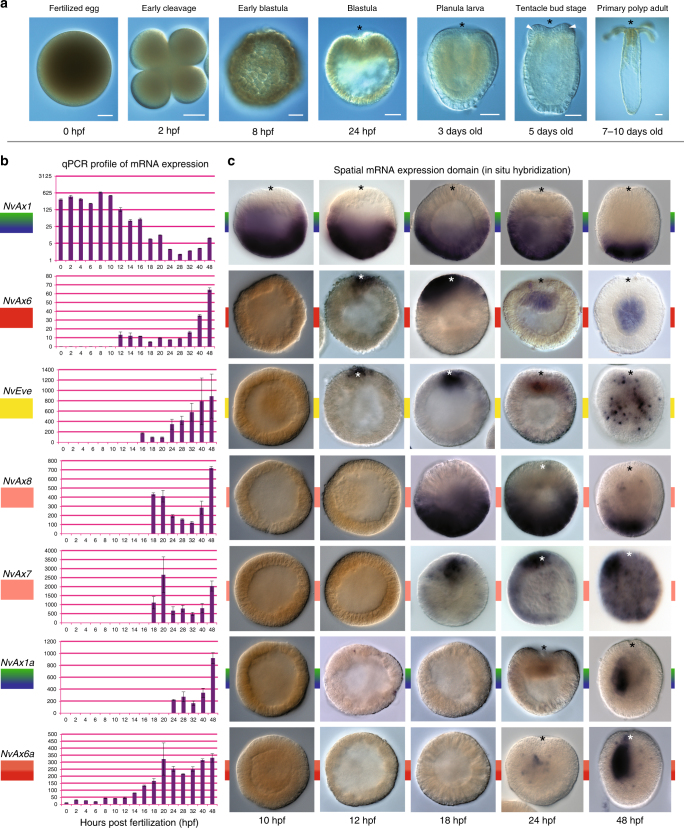
*Hox* genes of *Nematostella* exhibit hallmarks of bilaterian *Hox* genes during early development. **a** Time series of the embryonic, larval, and adult development of the cnidarian, *Nematostella vectensis* (hours post fertilization = hpf). **b** Clustered *Hox* genes display temporal colinearity during early embryonic development. Expression begins with the 3′ located neighboring non-*Hox* homeobox gene *NvHlxB9* (data not shown) and the anterior gene *NvAx6* at 12hpf. Subsequent activation of the other genes in the cluster maintains colinear expression relative to the ancestral cnidarian cluster. Two of the unlinked *Hox* genes *NvAx1* (central/posterior) and *NvAx6a* (anterior) appear maternally expressed during early development**. c** In situ hybridization confirms the temporal activation of the *Hox* cluster. Prior to gastrulation, mRNA expression of the central/posterior *Hox* gene (*NvAx1*) localizes to the aboral pole, while an anterior *Hox* gene (*NvAx6*) becomes transcriptionally activated along the oral pole. Remaining *Hox* and homeobox genes occupy oral (*NvEve*,* NvAv7*,* NvAx6a*) or aboral (*NvAx8*) domains during early development. Images were compiled from at least three separate experiments. Error bars are based on S.E.M. Scale bars are 50 µm

We analyzed the expression of *Hox* and neighboring non-Hox homeobox genes in *Nematostella* throughout early development (Fig. 2a), using a highly sensitive method (quantitative PCR) and verified expression by in situ hybridization. mRNA of both anterior and central/posterior *Hox* genes were detected early in development (Fig. 2b). Maternal mRNA of *NvAx1* and *NvAx6a* could be detected by qPCR in fertilized eggs (Fig. 2b, 0hpf); however, transcriptional activation of the extended *Hox* gene cluster begins during early blastula formation (Fig. 2b, 12hpf) with expression of the anterior *Hox* gene *NvAx6*. Deployment of the *Hox* cluster continues during the blastula to gastrula transition, with the activation of *NvEve* (Fig. 2b, 16hpf) and two genes related to *Hox*2 (*NvAx7* and *NvAx8*) (Fig. 2b, 18hpf). Lastly, activation of the central/posterior *Hox* gene (*NvAx1a*), which is fragmented from the predicted ancestral cnidarian *Hox* gene cluster (Fig. 1d)^22^, begins near the onset of gastrulation (Fig. 2b, 24hpf).

**Fig. 3:**
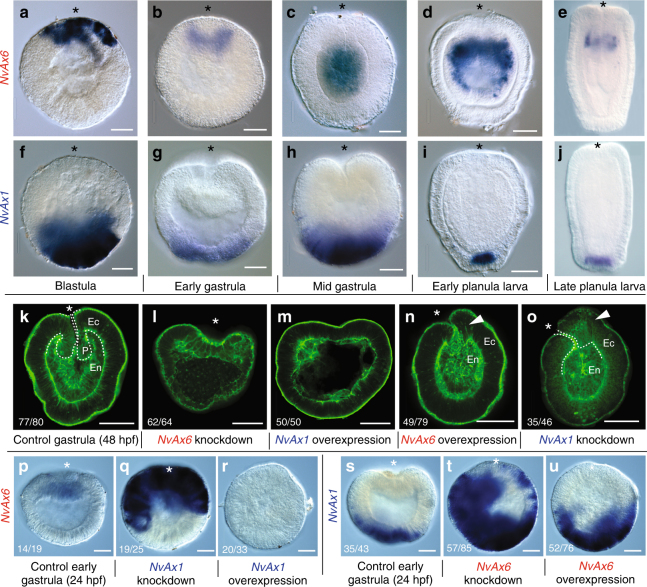
Perturbation of *Hox* expression disrupts oral patterning during gastrulation. Developmental time series of anterior, *NvAx6* (**a**–**e**), and central/posterior, *NvAx1* (**f**–**j**), *Hox* mRNA expression at blastula (**a**, **f**), early gastrula (**b**,** g**), mid gastrula (**c**, **h**), early planula (**d**,** i**), and late planula stages (**e**, **j**; images reproduced from ref^15^.). **k**–**o** Gastrulation defects due to disruption of anterior (*NvAx6*) and central/posterior (*NvAx1*) *Hox* expression through microinjection of antisense translation blocking morpholinos (knockdown) or in vitro transcribed mRNA (overexpression). **k** Fluorescent phalloidin-labeled embryo during final stages of gastrulation (48hpf) with distinct ectodermal(Ec) and endo-mesodermal(En) tissue layers delineated by the early pharynx (P) (white-dashed line delineates the pharynx from endomesoderm). **l** Knockdown of anterior *Hox* (*NvAx6*) blocks invagination of the presumptive endomesoderm. **m** Overexpression of central/posterior *Hox* (*NvAx1*) mRNA also blocks gastrulation and produces gastrula stage embryos with reduced axial morphology. **n** Overexpression of anterior *Hox* (*NvAx6*) mRNA and **o** knockdown of the central/posterior *Hox* gene (*NvAx1*) disrupts pharynx development and produces ectopic oral tissue at the blastopore (white arrowhead). **p**–**r** In situ hybridization of anterior *Hox (NvAx6*) at 24hpf in control (**p**), central/posterior *Hox* knockdown (**q**), and central/posterior *Hox* overexpression treatments (**r**). **s**–**u** In situ hybridization of central/posterior *Hox* (*NvAx1*) at 24hpf in control (**s**), anterior *Hox* knockdown (**t**), and anterior *Hox* overexpression treatments (**u**). Images were compiled from at least three separate experiments and the number of similar phenotypes is noted as a fraction in the lower left-hand corner (e.g., **k**—77 out of 80 total animals exhibited the phenotype). Scale bars are 50 µm

Two zones of spatial activation were found, with genes being expressed in oral (*NvAx6*, *NvAx6a*, *NvEve*, *NvAx7*,) or aboral (*NvAx1*, *NvAx8*) domains before and during early gastrulation prior to any asymmetries along the directive axis (Fig. 2b). Notably, *NvAx6* and *NvAx1* were the first to be detected by in situ hybridization, occupying complimentary oral and aboral domains during early blastula formation (12hpf) (Fig. 2c). *NvHlxB9* and *NvEve* are expressed along the oral pole of the animal during early gastrulation^35^, and in agreement with the qPCR analysis (Fig. 2b), the *NvHox* genes appear to be expressed along a similar temporal timeline. Respective oral and aboral expression of *NvAx6* and *NvAx1* continues through the blastula to gastrula transition. At this stage *NvAx6 is* initially expressed at the site of gastrulation (animal pole) in the presumptive endomesoderm before becoming restricted to the pharyngeal endoderm (Fig. 3a–e) associated with the pharyngeal nerve ring^15,20^. Conversely, *NvAx1* maintains a broad aboral expression domain throughout blastula and gastrula stages before becoming refined to the most aboral domain of the apical tuft region during early planula stages (Fig. 3f–j). The broad complimentary expression domains of the orally expressed anterior *Hox* gene *NvAx6* and aborally expressed *NvAx1* genes during blastula stages suggests they may play an important role in oral–aboral patterning during early development.

### Unequal opposing role of *Hox* genes in oral–aboral patterning

*NvAx6* and *NvAx1* are expressed in opposite territories throughout development and their function was manipulated by microinjection of mRNA or antisense oligonucleotides into uncleaved zygotes. Knockdown of the orally expressed anterior *Hox* gene, *NvAx6*, results in embryos that have defective gastrulation, compared with controls (Fig. 3k), and only initially form an inner endomesodermal plate (Fig. 3l). Overexpression of the aborally expressed *Hox* gene, *NvAx1*, by mRNA microinjection, results in a loss of gastrulation (Fig. 3m). Overexpression of the anterior *Hox* gene *NvAx6* or morpholino knockdown of *NvAx1*, each interfere with pharyngeal development and produce external tissue with a pronounced asymmetry at the blastopore that fails to invaginate (Fig. 3n, o). At early gastrula stages (24hpf), knockdown of the orally expressed *Hox* gene, *NvAx6*, and the aborally expressed *Hox* gene, *NvAx1*, caused an expansion of *NxAx1* and *NvAx6* expression respectively (Fig. 3p–q, s, t). However, overexpression of the anterior *Hox* gene *NvAx6* exhibits only a slight expansion of *NvAx1* expression (Fig. 3u). Conversely, overexpression of aboral *Hox* gene, *NvAx1*, completely abolishes *NvAx6* expression at the oral pole (Fig. 3r) and inhibits oral development (Fig. 3m). This suggests that *NvAx6* and *NvAx1* maintain respective oral and aboral expression domains through antagonism, but that *NvAx1* appears to have functional dominance over *NvAx6*, or that other factors contribute to the lack of antagonism in *NvAx6* overexpression experiments (Fig. 3u).

**Fig. 4:**
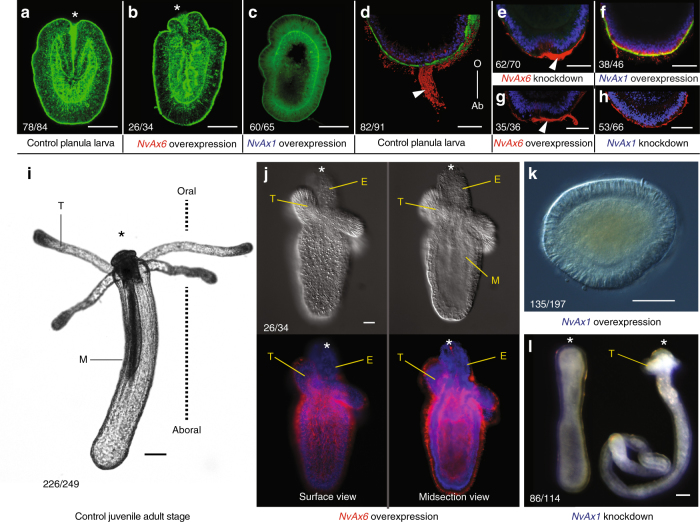
Effects of perturbing anterior and central/posterior *Hox* genes on oral–aboral identity extends to late development. **a**–**c** Phalloidin stained embryos at planula larvae (144hpf) stages taken from: **a** dextran-injected controls, **b** anterior *Hox* (*NvAx6*) overexpression, and **c** central/posterior *Hox* (*NvAx1*) overexpression treatments. **d**–**h** Apical tuft cilia labeled with and acetylated tubulin antibody (red) with the nuclei counter stained with DAPI (blue) in: **d** planula stage embryos treated with dextran, **e** anterior *NvAx6* morpholino, **f** central/posterior *NvAx1 Hox* mRNA, **g** anterior *NvAx6* mRNA, or **h** central/posterior *NvAx1 Hox* morpholino. **i**–**l** Changes in polyp morphology resulting from disruption of anterior *Hox* (*NvAx6*) and central/posterior *Hox* (*NvAx1*) expression during early development. **i** Wild-type polyp after metamorphosis. **j** Overexpression of anterior *Hox* (*NvAx6*) results in ectopic tissue formation at the oral side of the animal. **k** Overexpression of central/posterior *Hox* (*NvAx1*) results in bilayered animals (maintained for 21 days after fertilization) without clear axial morphology but have cnidocytes (stinging cells) along the outer ectoderm. **l** Animals treated with central/posterior *Hox* (*NvAx1*) morpholino eventually metamorphose (2 weeks after fertilization) to produce animals with small heads and extremely long body columns. Images were compiled from at least three separate experiments and the number of similar phenotypes is noted as a fraction in the lower left-hand corner. Scale bars are 50 µm

The morphological changes found during early development and gastrulation are consistent with phenotypes identified during larval and polyp formation (Fig. 4). Three days after injection, ectopic tissue around the larval mouth is still present in animals with overexpression of *NvAx6*, where overexpression of *NvAx1* inhibits larval development (Fig. 4a–c). The aboral sensory apical tuft is only affected by overexpression or knockdown of *NvAx1* (Fig. 4d–h). Finally, overexpression of *NvAx6* results in ectopic tissue also in the juvenile polyp, consistent with expansion of oral territories created during gastrulation. *NvAx1* overexpression blocks polyp formation (Fig. 4k), where knockdown of *NvAx1* results in deformed polyps with elongated body columns with less oral features, such as tentacles (Fig. 4l).

**Fig. 5:**
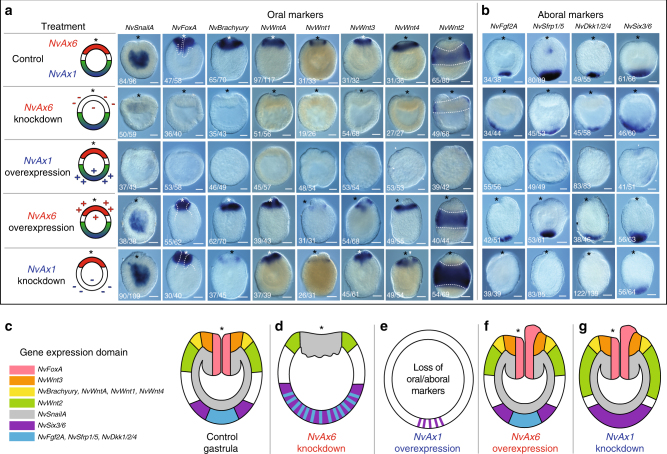
Anterior and central/posterior *Hox* genes have reciprocal phenotypes during oral but not aboral development. **a** Schematic representation of perturbation of either anterior *Hox* (*NvAx6*) or central/posterior *Hox* (Ax1) during early development. **a**, **b** Late gastrula (48hpf) stage expression of molecular markers for oral (**a**) or aboral (**b**) territories. White-dashed lines in *NvFoxA* column serve to highlight the larval pharynx and distinguish between the ectoderm and endoderm. White-dashed lines in the *NvWnt2* column outline the belt of *NvWnt2* expression. **c**–**g** Illustration summarizing territorial changes due to manipulation of anterior (*NvAx6*) and central/posterior (*NvAx1*) *Hox* genes. Images were compiled from at least three separate experiments and the number of similar phenotypes is noted as a fraction in the lower left-hand corner. Scale bars are 50 µm

These findings suggest that altering the *Hox* program during early development results in changes along the oral–aboral axis that are maintained throughout development. To understand what changes occur as a result of manipulation of *Hox* expression, we analyzed a diverse set of developmental patterning genes and their relationship to *NvAx6* and *NvAx1* during early development. Control injections of dextran or control morpholinos do not impair expression of any genes analyzed herein (Supplementary Fig. 2). The endomesodermal marker, *NvSnailA*, is reduced in *NvAx6* knockdown experiments and is lost in *NvAx1* overexpression treatments (Fig. 5a, c–e). In these two treatments, embryos continue to gastrulate (Fig. 3l–m), fail to form the pharynx, and do not express the pharyngeal marker *NvFoxA* or the oral marker *NvBrachyury* that is expressed at the ectodermal/pharyngeal boundary (Fig. 5a), and which has been shown to regulate pharynx formation in anthozoans^36,37^. *NvAx6* knockdown shows minor expansion of aboral markers such as *NvFGF2A*, *NvSfrp1/5*, *NvDkk1/2/4*, and *NvSix3/6* (Fig. 5b), but does not appear to effect aboral development, as embryos treated with *NvAx6* anti-sense morpholino eventually form a swimming planula larva with an apical tuft at the aboral pole (Fig. 4e). Surprisingly, *NvAx1* overexpression reduces aboral markers expression (Fig. 5b–c, e) and the apical tuft in this region does not form (Fig. 4f). *NvSix3/6* appears both diminished and disorganized, expanding into multiple regions of the embryo (Fig. 5b–c, e).

Overexpression of the anterior *Hox* gene *NvAx6* or morpholino knockdown of *NvAx1* produce ectopic tissue that expresses the pharyngeal/oral markers *NvFoxA* and *NvBrachyury* (Fig. 5a, c, f, g). *NvSnailA* is expressed normally in cells of the endomesodermal plate indicating that these defects are related to pharyngeal patterning and not endomesoderml plate specification (Fig. 5a). Aboral markers are lost in *NvAx1* knockdown treatments, suggesting that the *NvAx1* expression is necessary for aboral specification (Fig. 5b–c, g). However, aboral marker expression and apical tuft formation are not disturbed by *NvAx6* overexpression (Figs 4g, 5b, c, f).

### Role of *NvAx6* and *NvAx1* patterning during early development

**Fig. 6:**
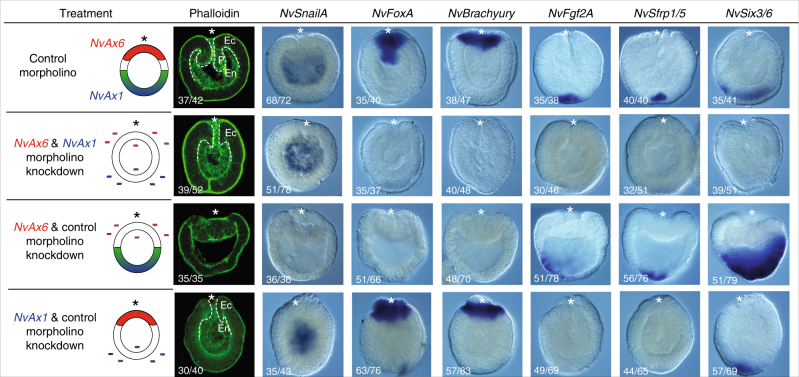
Loss of anterior and central/posterior *Hox* results in loss of oral–aboral patterning, but not endomesoderm formation. Gastrulation defects resulting from co-injection experiments as assessed by fluorescent phalloidin labeling (far left column) and in situ hybridization for oral (*NvSnailA*,* NvBrachyury*, and *NvFoxA*) and aboral (*NvFgf2a*, *NvSfrp1/5*, and *NvSix3/6*) markers. Ec ectoderm; En endo-mesodermal; and P pharynx. White-dashed line serves to distinguish the point of transition between the ectoderm and the endoderm. Images were compiled from at least three separate experiments and the number of similar phenotypes is noted as a fraction in the lower left-hand corner

Disruption of oral or aboral *Hox* genes expand opposing territories (Fig. 3q, t). Ectopic aboral *Hox* (*NvAx1*) expression restricts gastrulation and oral specification, resulting in the lack of endomesoderm formation throughout development (Fig. 5a). Similarly, knockdown of the oral *Hox* gene *NvAx6* produces a severe defect in gastrulation (Fig. 3l) and results in an expansion of *NvAx1* expression toward the oral pole (Fig. 3t). This suggests that stalled gastrulation as a result of *NvAx6* knockdown is, in part, a result of the upregulation and expansion observed by the *NvAx1* transcript, which antagonizes oral development (Fig. 3m). To test this hypothesis, we co-injected both *NvAx6* (oral) and *NvAx1* (aboral) morpholinos into *N. vectensis* zygotes and assessed changes in late gastrulae (48hpf). Controls were injected with either *NvAx6* or *NvAx1* morpholinos with a standard control morpholino and produced phenotypes identical to those seen with normal single gene-specific morpholino injections (Fig. 6). Embryos treated with both *NvAx6* and *NvAx1* morpholinos undergo gastrulation to form an outer ectoderm (Ec) and an inner endomesoderm (En) that expresses the maker *NvSnailA*, but fail to form a pharynx(P) or express the pharyngeal markers *NvFoxA and NvBrachyury* (Fig. 6). Aboral markers assessed in this study were lost in double injection (*NvAx6* + *NvAx1*) experiments, including a complete loss of *NvSix3/6* (Fig. 6), which is reduced but not lost in *NvAx1* morpholino treatments. Co-injection of *NvAx6* and *NvAx1* morpholinos has no effect on oral marker expression, including the pharyngeal marker *NvFoxA* at earlier gastrula stages (24hpf) (Supplementary Fig. 3), suggesting that patterning cues downstream of the oral *Hox* gene, *NvAx6*, are required after the onset of gastrulation. However, the aboral marker *NvSix3/6* is lost at 24hpf in treated embryos (Supplementary Fig. 3), demonstrating that *NvAx1* expression has a patterning role at these earlier gastrula stages.

### *NvAx6* and *NvAx1* regulate oral–aboral axis through Wnt

The oral–aboral axis of cnidarians is thought to be patterned by restricted Wnt domains beginning at the onset of gastrulation and progressing through larval development^38–43^. *NvWnt1*, *NvWnt3*, *NvWnt4*, and *NvWntA* are expressed around the blastopore/oral pole and disappear following both oral *Hox NvAx6* knockdown and aboral *Hox NvAx1* overexpression (Fig. 5a). *NvWnt2* is expressed in a band along the ectodermal midline of the embryo and serves as an important marker for defining oral and aboral territories. Knockdown of *Hox NvAx6* expression results in shift of *NvWnt2* expression toward the oral pole, while overexpression of *NvAx6* has no observable effect on *NvWnt2* expression (Fig. 5a). *NvWnt2* expression is lost in aboral *Hox* (*NvAx1*) overexpression treatments, while knockdown of *NvAx1* resulted in a robust upregulation and aboral expansion of the *NvWnt2* domain (Fig. 5a). A more pronounced phenotype was produced in CRISPR cas9-mediated knockout of *NvAx1*, resulting in upregulation of *NvWnt2* throughout the entire ectoderm and endomesoderm (Supplementary Fig. 4). *NvAx1* knockouts also expanded the orally expressed *NvWnt1* into the endomesoderm (Supplementary Fig. 4). These findings suggest that *NvWnt2* is the intermediary between the ectodermal oral–aboral *Hox* gene boundary and that territorial Wnt expression is mediated by blastula *Hox* patterning.

## Discussion

Since the first cnidarian genome became available^44^ it has become clear that much of the developmental “toolkit” deployed during the development of bilaterian species remains present in the cnidarian lineage. Although there are aspects of cnidarian *Hox* clusters that have diverged from the ancestral condition in virtually all lineages (e.g., loss or duplication of *Hox*2 homologs, diversification of central/posterior genes in *Hydra*, Supplementary Fig. 1), it appears that the common ancestor of cnidarians and bilaterians utilized *Hox* genes to pattern their primary (oral–aboral) body axis. If a *Hox* cluster is representative of the ancestral condition in Cnidaria, gene duplications and loss may have played an instrumental role in the divergent life history evolution (e.g., metagenic lifecyles) within the phylum. For example, the hydrozoan cnidarian, *Clytia hemisphaerica*, has a medusa (jellyfish) life history stage and very similar set of *Hox* genes with *Nematostella*^21^. However, comparison of the ancestral role of *Hox* genes in these two species are difficult to make at this time because the genomic organization of *Hox* genes in *Clytia* is unresolved, and similar to early work in *Nematostella*^15,20^, expression analysis by in situ hybridization may only have highlighted stages exhibiting elevated levels of mRNA. Additionally, no experiments have investigated the embryonic patterning role of *Hox* genes in any other cnidarian. Previous studies in *Hydra*, which lacks both the larva and medusa lifecycle stages^45,46^, has highlighted the role of *Hox* genes in patterning the adult polyp oral–aboral axis^31,33,34^. It will be interesting to see if gene loss (e.g., loss of *Eve* in *Hydra*) and cluster fragmentation helped shape the diverse life history stages and embryonic development of cnidarians.

There exists a longstanding argument about the origin of axial patterning machinery outside of bilaterians. Our results indicate that anterior and central/posterior *Hox* genes play important roles specifying oral and aboral territories, respectively, prior to the onset of gastrulation. During gastrulation and on through planula development, a complex developmental program is initiated that involves Wnt/β-catenin signaling that patterns the oral–aboral axis. During this time, Bmp agonists/antagonists activate *Hox* genes in the endomesoderm layer along a second axis, perpendicular to the oral–aboral axis, called the directive axis (may or may not be homologous to the dorsal–ventral axis of bilaterians)^47–49^. The directive axis is likely established around the onset of gastrulation, after establishment of the oral–aboral axis at the site of gastrulation. If the evolutionary origins of distinct clades of *Hox* genes (e.g, anterior class *Hox* genes) pattern similar regions of cnidarian body plans, as they do in bilaterians (i.e., anterior body regions), our findings would argue that the oral pole is homologous to the bilaterian anterior pole and the posterior end would be equivalent to the cnidarian aboral pole. This is consistent with numerous studies that show the cnidarian animal pole gives rise to the oral pole^50–54^, just as it does in virtually all other bilaterians^55–58^ and in another early branching basal animal lineage, the ctenophores^59,60^. If the phylogenetic relationship of these genes also coincides with axial positioning, these findings would suggest that the oral–aboral axis is homologous to the bilaterian A–P axis, with the adult mouth corresponding to anterior in both cases. The alternatives are that A–P positioning never existed prior to bilaterians^13^ or it could be argued that early animals may have possessed only “anterior-like” *Hox* genes without true central/posterior class genes^14^. The later scenario appears to be less likely, as medusazoan cnidarians like *Clytia* and *Hydra* appear to have true central/posterior class genes^21^, although greater taxonomic sampling is needed. Despite what we know about *Hox* genes in cnidarians, some components of the molecular patterning of the apical tuft neurosensory cilia region appear to be similar in different invertebrate larvae^61–63^. Further characterization of this transient larval apical tuft region across cnidarians is warranted, because some of the most robust indicators like the homeobox gene, *Six3/6*, is expressed orally in other cnidarians^64^. Of course, additional studies are required to determine with certainty the molecular basis for axial development in animal evolution.

**Fig. 7:**
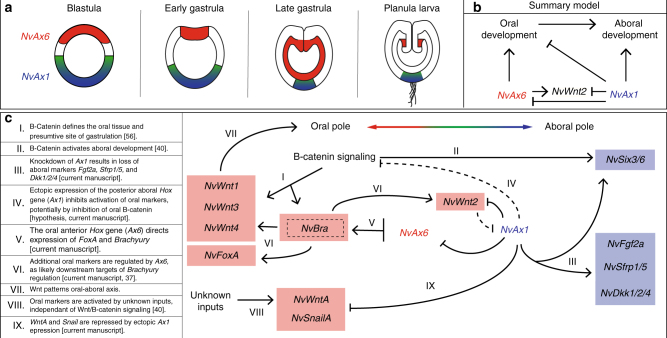
Model of *Hox* gene function in early development of an anthozoan. **a** Illustration depicting anterior *Hox* (*NvAx6*) (red) and central/posterior *Hox* (*NvAx1*) (blue/green) expression throughout early development of *N. vectensis*. **b** Working model of how anterior (*NvAx6*) and central/posterior (*NvAx1*) *Hox* genes function to specify the oral–aboral axis before and during gastrulation in an anthozoan cnidarian. **c** Illustration of known regulatory relationships between *Hox* genes and the oral (*NvWntA*, *NvWnt1*, *NvWnt2*, *NvWnt3*, *NvWnt4*, *NvFoxA*, *NvBrachyury*, and *NvSnailA*) and aboral (*NvSix3/6*, *NvFgf2A*, *NvSfrp1/5*, and Nv*Dkk1/2/4*) markers assessed in this study. Important components of this regulatory paradigm needing description are marked with roman numerals (I–IX) with corresponding text at the periphery of the illustration. Regulatory relationships derived from previous studies are cited in the periphery text (I, II, VI, and VIII)

In this report, we have characterized for the first time in any cnidarian embryo the existence of early domains of reciprocal *Hox* gene expression. In the starlet sea anemone, *Nematostella vectensis*, an anterior *Hox* gene is expressed at the beginning of blastula stages, extending through gastrulation and functionally interacts with a maternally expressed central/posterior *Hox* gene in oral–aboral axis formation and tissue specification. Although suggested to be primarily a vertebrate phenomenon^65^, elements of the hierarchal prevalence among *Hox* genes appears to be functioning in *Nematostella*. *NvAx6* and *NvAx1* act in a partial antagonistic fashion to pattern both oral and aboral territories (Fig. 7a, b), which appears to be mediated through interactions with Wnt signaling. Through these studies, we can begin to understand the molecular basis of oral–aboral patterning in *Nematostella* and develop a working model of cnidarian *Hox* function (Fig. 7c).

During early cleavage stages, the stabilization of β-catenin and components of both canonical and PCP Wnt signaling pathways establish the site of gastrulation/future oral pole in *Nematostella*^66,67^ (Fig. 7c; I). Recent findings have shown that *Nvβ-catenin* activity is upstream of *NvBrachyury*^50^ and that *NvBrachyury* is upstream of Wnt signaling^51^ at the oral pole, a process that is partially mediated by the homeobox gene *NvSix3/6*^68^ (Fig. 7c; II). Our findings suggest that the maternal aboral marker *NvAx1* also suppresses activation of Wnt signaling at the oral pole, when overexpressed, potentially through blocking activation of *NvAx6* and downstream targets (e.g., *NvBra*, *NvWntA*, *NvWnt1*, *NWnt3*, and *NvWnt4*) (Fig. 7c; IV). These results are consistent with crosstalk between *Nvβ-catenin* and aboral patterning^68^, where oral *Nvβ-catenin* is necessary to initiate aboral patterning. Therefore, the loss of aboral genes as a result of *NvAx1* overexpression may be a result of the inactivation of oral *β-catenin*, in turn effecting both oral and aboral specification. Recent work began to establish the gene regulatory network driving endomesoderm formation in *Nematostella*, and highlighted the blastopore ring domains along the oral/animal pole^35^. Our findings add greater detail to this network and suggest that the *NvWnt2* expression domain could be the boundary region between dedicated oral–aboral territories. Hyper activation of *β-catenin* signaling through pharmacological treatment was shown to shift *NvWnt2* boundaries toward the aboral pole, while suppressing *NvAx1*^43^. *Nvβ-catenin* knockdown abolishes *NvWnt2* expression^68^ yet the functional link with oral and aboral *Hox* genes has yet to be resolved.

The formation of drastically elongated polyps in aboral *Hox* (*NvAx1)* knockdown (Fig. 4l) points to a captivating similarity between the functions of vertebrate and anthozoan central/posterior (and posterior-like) *Hox* genes in axial elongation. Similar phenotypes were also witnessed as a result of mis-regulation of *Nvβ-catenin* signaling^66^, providing greater evidence for a link between these two molecular pathways. Results from the double knockdown of *NvAx6* and *NvAx1* provides support for the proposed role of *NvAx1* in restricting oral specification, possibly through the restriction of Wnt signaling, as parallel knockdown of *NvAx1* was sufficient to rescue the loss of gastrulation and oral specification phenotype produced in *NvAx6* morpholino treatments. From these results, we propose that *NvAx1* is an inhibitor of Wnt/β-catenin signaling (Fig. 7c; IV) similar to what has recently been described during vertebrate development^69^. Therefore, the loss of *NvAx1* expression in oral territories is necessary for establishment and progression of oral patterning and the core regulatory relationship between central/posterior (posterior-like) *Hox* expression and Wnt signaling may be a highly conserved characteristic of posterior *Hox* function. Further investigation into the mechanism of this relationship and the hierarchal nature of the other *NvHox* genes will help determine if the paradigm of posterior dominance is truly conserved between bilaterians and cnidarians, and will help resolve if *NvAx6* exhibits any form of anterior dominance.

The phylogenetic origin of the *Hox* patterning system is a highly controversial matter among evolutionary developmental biologists. The cnidarian clade has the greatest diversity of *Hox* genes among the four basal animal lineages (sponge and ctenophore genomes do not contain *Hox* genes^25,26,28^). Genomic resources from a diverse group of cnidarians suggest rearrangements and fragmentations of genomic clusters are present among cnidarian species. The axial patterning role of cnidarian *Hox* genes was long debated^21,31–34^, yet the functional analysis of *Hox* genes in *Nematostella* indicate that some aspects of genomic organization and A–P patterning were present in the cnidarian ancestor. It will be interesting if the study of other cnidarian species reveal an additional role for *Hox* genes during early development, which will ultimately help resolve the shared and derived characters of cnidarian and bilaterian *Hox* genes. Together these data suggest that cnidarians retain components of a simple ancestral *Hox* system that was originally deployed during early developmental stages and functioned to organize the primary axis in the cnidarian ancestor.

## Methods

### Animal care

Adult *Nematostella vectensis* were raised in 1/3× seawater at 16 °C in constant dark. Animals were fed artemia once a week and fed oyster 48 h before spawning, which was engendered by an 8-h light box cycle at 16 °C. Distinct groups of animals were spawned once every 3–4 weeks. Fertilized embryos were collected and placed in a 4% cysteine wash (4% cysteine in 1/3 filtered seawater, pH 7.4) for 15 min to remove the outer jelly layer. Eggs were then washed (3×) with 1/3 filtered seawater before conducting experiments. Treated embryos were raised to collection at 16 °C. Embryos used were from randomized individuals from different genetic individuals to eliminate genetic variability. Mixed genetic pools of individual animals created a blind test of the effectiveness of each treatment regardless of genetic ancestry.

### Microinjection knockdown overexpression and knockout

We disrupted *NvAx6* and *NvAx1 Hox* gene expression using gene-specific antisense translation blocking morpholinos or CRISPR/Cas9-mediated genome editing using multiple guide RNAs (gRNAs) against each gene^36,49^. Genes were over expressed by injecting in vitro transcribed mRNA created using mMessage in vitro transcription kits (Ambion AM1348). Morphological and molecular analysis of phenotypes was conducted during late gastrula (48hpf), planula larvae (96hpf) stages of development, and 1–2-week-old polyps. Control injections using a standard control morpholino and dextran lineage tracer (or Cas9 protein without gRNAs in knockout injections) produced normal gastrula stage embryos (Fig. 3k) possessing an outer ectoderm(Ec), inner endomesoderm(En), and pharynx(P) (dashed white line) (Supplementary Fig. 2). Knockdown and knockout treatments produced similar molecular phenotypes (Supplementary Fig. 4), thus validating the efficacy of the *NvAx6* and *NvAx1* morpholinos. Treated embryos were raised to collection at 16 °C.

Translation blocking morpholinos against *NvAx6* and *NvAx1* and a standard control morpholino have been developed through Gene Tools, LLC. Philomath OR, 97370. Morpholino sequences are as follows (Standard Control 5′-CCTCTTACCTCAGTTACAATTTATA-3′; *NvAntHox6* 5′-ACCGCCGCTCATGCCCAAATGTGTC-3′; *NvAntHox1* 5′-TTGACTGCATGATGTGCGCTCTAGT-3′). Expression constructs for *NvAx6* and *NvAx1* were generated using the gateway cloning system (SPE3-Ax1-Rvenus, SPE3-Ax6-RmCherry, and SPE3-Ax6a-RmCherry). Morpholinos and mRNA were first injected in a dilution series ranging in concentration from 0.1 and 0.9 mM and appropriate concentrations (0.3 mM for *NvAx6* MO, 0.5 mM *NvAx6* mRNA, 0.9 mM for *NvAx1*, and 0.3 mM *NvAx1* mRNA) were selected to achieve an optimal relationship between toxicity and phenotype penetrance.

CRISPR/Cas9 genome editing in *Nematostella* has recently become a useful tool for genetic manipulation^36,49^. To perform knockouts of *Hox* genes, target sequences 18–20 bp in length fitting the gRNA target profile of 5′-G(G-A)-N(16-18)-NGG-3′ were identified within the coding sequence of the *NvAx6* and *NvAx1* locus (Supplementary Figs 5a, 6a) using a web-based program called ZiFiT (http://zifit.partners.org). Six target sequences were selected for each locus bases on their location (choosing sites scattered throughout the coding sequence) and their gRNA efficiency score, which was calculated using the web-based CRISPR Efficiency Predictor provided by the DRSC at Harvard Medical School (http://www.flyrnai.org/evaluateCrispr/). Sequences and efficiency scores are listed in Supplementary Table 1. Additionally, potential target sequences were blasted against the publically available *Nematostella* genome (http://genome.jgi.doe.gov) to insure that there were minimal off-target sites related to the sequence, limiting partial off-target sites to having no more than 15 base pairs in common with the 18–20 base pair target sequence. Oligonucleotides were generated integrating the target sequence (5′-G(G-A)-N(16-18)-NGG-3′) into a CRISPR RNA (crRNA) sequence containing a T7 (5′- AATTAATACGACTCACTATA-3′) or Sp6 (5′-AATATTTAGGTGACACTATA-3′) promoter. Full-length gRNA was generated from a PCR assembled trans-activating crRNA (tracrRNA) oligonucleotide (Supplementary Table 1), followed by in vitro transcription (NEB Highscribe™ T7 and Sp6 RNA synthesis kits; Cat# E2040S and E2070S), and RNA purification (Zymogen RNA Clean and Concentrator™-25; Cat# R1017). gRNAs at a final concentration of 400 ng/µl (consisting of equal concentrations of each gRNA) were injected with Lyophilized bacterial type II Cas9 protein (PNA Bio, Thousand Oaks, CA) (1 μg/µl) reconstituted in 50% glycerol and 0.1 mM DTT to generate frame shift mutations and deletions within the target locus. Genomic DNA was isolated from 8 individual 18hpf embryos per treatment group for PCR analysis of the targeted region using a DNA extraction kit, according to the manufacturers protocol (Qiagen 69504) (Supplementary Figs 5b, 6b). Additionally, in situ hybridization was performed on treated blastula stage embryos to ensure that gene expression was lost (Supplementary Figs 5c, 6c). Treated embryos were collected at 48hpf for in situ hybridization studies to assess changes in marker gene expression (Supplementary Fig. 4).

### Quantitative PCR

Quantitative PCR was performed using the Roche LightCycler^®^ 480 Instrument II and LightCycler 480 SYBR Green I Master mix (Cat# 04707516001, Roche, Inc.). qPCR samples were standardized with *NvGADPH* and *NvRiboPro* and primers for other genes were designed using MacVector (www.macvector.com) to amplify 75–150 base-pair fragments of the desired gene. These primers were then back-blasted against the *Nematostella* genome to make sure they only will amplify a single region from the genome. We checked each primer efficiency with a dilution curve (10^−1^–10^−5^) to make sure their range was within the negligible value of 1.9–2.0. Individual stages were collected from pooled samples of embryos consisting of roughly 100 embryos. These stages represent a single biological sample, and were confirmed through technical replication. Total RNA from each sample was stored in TRIzol (15596-026) at −80 **°**C until processed. RNA was isolated according to manufacturer’s specification. RNA was DNase treated using Qiagen RNase-free DNase #79254 for 15 min at 37 °C. cDNA was standardized by using 1 µg of total RNA and synthesis was conducted using the Advantage RT-PCR kit (Clontech, 639506) for qPCR analysis. Samples were run using a Roche LightCycler 480 qPCR platform. Roche SYBR Green 1 Master mix (Roche 04887352001) was used for all qPCR. Relative fold change values were calculated in Microsoft Excel.

### In situ hybridization

All in situ hybridizations were conducted as described below. Fixations were done in 1% gelatin-coated dishes to prevent tissue from sticking to the plastic (sticking to plastic causes tissue damage and non-specific staining). Embryos were fixed in ice cold 4% paraformaldehyde with 0.2% glutaraldehyde in 1/3× seawater for 2 min, followed by 4% paraformaldehyde in 1/3× seawater for 1 h at 4 °C. Embryos were stored in 100% methanol at −20 °C until used. Samples were rehydrated through a methanol series (75% methanol/25% PBS + 0.1% Tween 20, 50% methanol/50% PBS + 0.1% Tween 20, 25% methanol/75% PBS + 0.1% Tween 20) and then washed 3× in PBS + 0.1% Tween 20 (Ptw). Embryos were treated with 0.1 mg/ml Proteinase K in Ptw for 5 min, followed by 2× washes in Ptw + 2 mg/ml glycine. Embryos were moved to 24-well tissue culture plates, then incubated in 0.1% triethanolamine in Ptw for 5 min, followed by two washes with 0.1% triethanolamine in Ptw + 3 µl/ml of acetic anhydride. Samples were then washed 2× in Ptw, followed by a 1 h fixation in 4% PFA in Ptw at room temperature. Afterward samples were washed 5× in Ptw and then incubated overnight in hybridization buffer.

Probe sequences ranging from 550–1200 bps were cloned from cDNA using the pGEM^®^-T vector system and DIG-labeled RNA probes were generated using MEGAscript in vitro transcription kits (Invitrogen, AM1333M). If available, cloned marker gene sequences were selected from a probe stock originally created for a previous study^35^. Probes were hybridized at 64 °C for 2 days and developed with the enzymatic reaction of NBT/BCIP as substrate for the alkaline phosphatase-conjugated anti-DIG antibody (Cat# 11093274910, Roche, Inc.). Wild-type samples were developed for an equal amount of time and if no expression was visible, a subset of samples remained in developing solution for at least 1 day to determine if lower levels of expression was present. When developing in situs of treated embryos, development time was based on the signal seen in control samples. All experiments exhibited greater than 50% penetrance of phenotypes, with 75% of the data containing greater than 75% penetrance. Due to variability during injection, embryo size and development time of different genes, greater than 50% in replicates, were considered the dominant phenotype created by genetic manipulations. Furthermore, to obtain material to test all the genes, samples were collected from multiple days of injection and pooled for in situ hybridization. Replicate attempts of in situ hybridization were attempted at least twice on pooled samples to obtain sample sizes generally greater than 30 embryos per replicate.

### Immunostaining

Methods for immunostaining were adapted from previously published protocols^51^. Embryos collected for immunostaining were fixed in ice cold 4% paraformaldehyde with 0.2% glutaraldehyde in 1/3× seawater for 2 min, followed by 4% paraformaldehyde in 1/3× seawater for 1 h at 4 °C. Samples were then washed five times (5 min each) in PBT (1% Triton-x and 1% BSA in PSS) and stored in PBS at 4 °C for up to 1 month. Samples were then washed three times (30 min each) in PBT followed by a 1-h block in blocking solution (5% normal goat serum in PBT) at room temperature. Samples were then treated with the primary antibody (monoclonal anti-α-Tubulin; SigmaT9026) diluted 1:500 in blocking solution and incubated overnight (12 h) at 4 °C. Primary antibody was removed and samples were rinsed five times (10 min each) in PBT before adding the secondary antibody (goat-anti-mouse-568; A-11004) diluted 1:250 in blocking solution and incubated over night at 4 °C. Tissues were then washed at least three times (15 min each) in PBT before adding counterstain. DAPI was used (0.1 μg/µl in PBS) was used to label cell nuclei. Fluorescent phalloidin (Invitrogen A12379) was used at a concentration of 1:200 in PBT to stain filamentous actin, incubating for no more than 3 h at room temperature. Samples were washed at least three times (15 min each) in PBS before clearing. Samples were cleared in 80% glycerol or using Murray’s Clear (1:1 benzyl benzoate and benzyl alcohol).

### Microscopy and imaging

Results from in situ hybridization studies were imaged using a Zeiss Axio Imager Z1 with a Zeiss HRc color digital camera run by Zeiss Zen 2012 software. Fluorescently labeled embryos were imaged using a Zeiss LSM-710 confocal microscope. Images of polyp stage results and whole wells of 24-well plates (as pictured in Fig. 4 and Supplementary Figs 5–6) were imaged using A Zeiss SteREO Discovery.V8 stereoscopic microscope with a Cannon EOS 5D MarkII full frame DSLR camera. Images were processed and scale bars were added using the Fiji distribution of ImageJ (http://fiji.sc).

### Data availability

The authors declare that all data supporting the findings of this study are available within the article and its Supplementary Information files or from the corresponding author upon reasonable request. All figures and supplementary data are available through FigShare (10.6084/m9.figshare.5900887). All primers used for generation of gRNA for CRISPR-Cas9 are available in Supplementary Table 1. Full-length transcripts used for probe production, mRNA misexpression, and designing morpholinos are available in the Supplementary Table 1. All protocols can be requested through Nature Protocol Exchange under the Martindale Whitney Lab group.

## Electronic supplementary material

Supplementary Information
