# Supplementary material for: Hox and Wnt pattern the primary body axis of an anthozoan cnidarian before gastrulation
Source: Nat Commun. 2018 May 22;9:2007. doi: 10.1038/s41467-018-04184-x (PMC5964151; doi:10.1038/s41467-018-04184-x)
Supplement: Supplementary file 1 — Supplementary Information [file 41467_2018_4184_MOESM1_ESM.pdf]

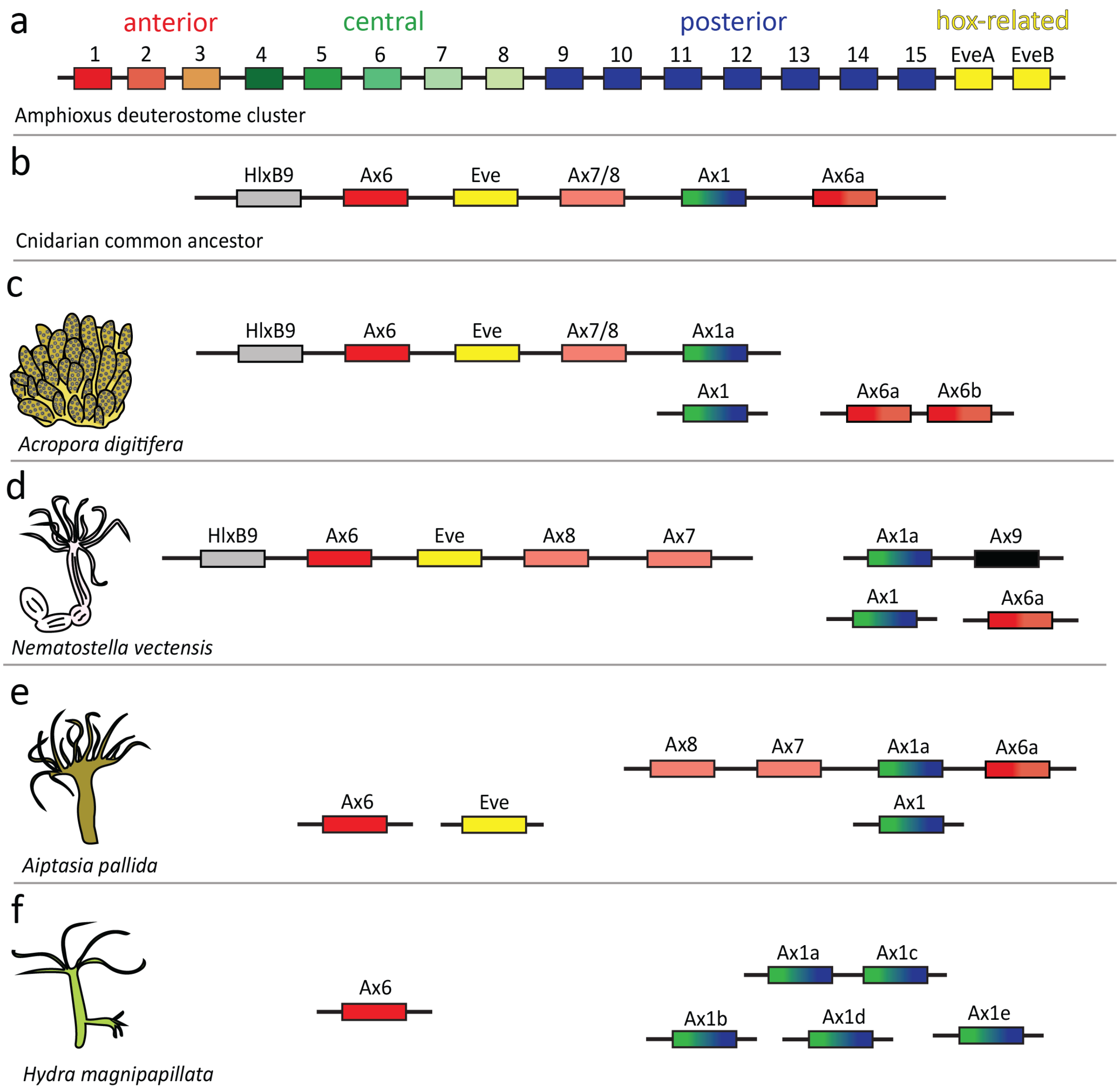

Supplementary Figure 1 Current view of *Hox* clustering found with cnidarian genomes
(a) Cluster of *Hox* genes found in the deuterostome, Amphioxus[1]. **(b)** *Hox* cluster of the proposed common ancestor of Cnidaria. **(c)** *Hox* cluster of *Acropora digitifera*[2]. **(d)** *Hox* cluster of *Nematostella vectensis*[3,4]. **(e)** *Hox* cluster of *Aiptasia pallida*[5]. **(f)** *Hox* cluster of *Hydra magnipapillata*[3].

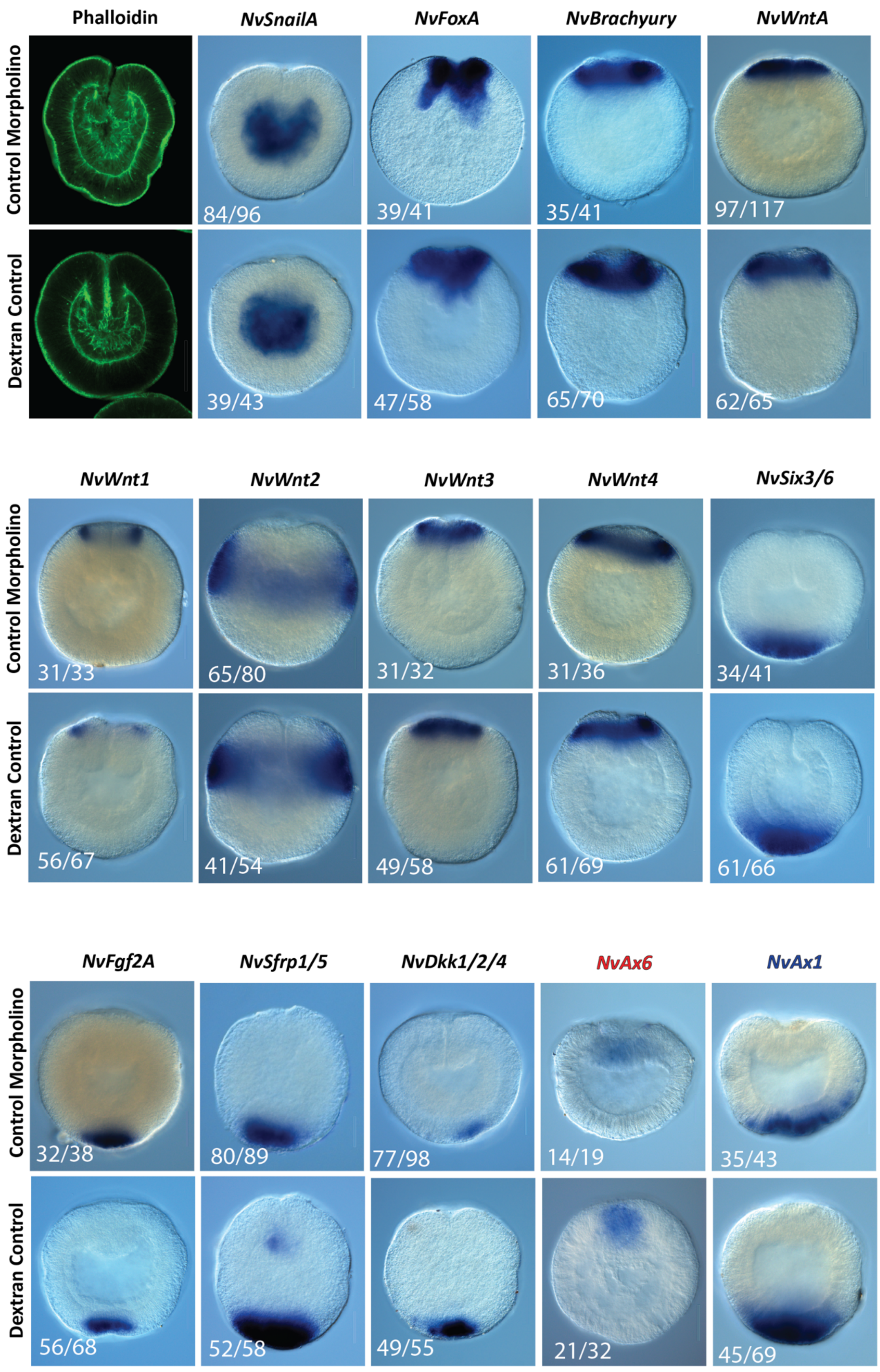
Phalloidin
NvSnailA
NvFoxA
NvBrachyury
NvWntA
Control Morpholino
Dextran Control
84/96
39/41
35/41
97/117
39/43
47/58
65/70
62/65
NvWnt1
NvWnt2
NvWnt3
NvWnt4
NvSix3/6
31/33
65/80
31/32
31/36
34/41
56/67
41/54
49/58
61/69
61/66
NvFgf2A
NvSfrp1/5
NvDkk1/2/4
NvAx6
NvAx1
32/38
80/89
77/98
14/19
35/43
56/68
52/58
49/55
21/32
45/69

Supplementary Figure 2 Results of control morpholino and dextran control treatments on gastrulation and axis specification

Wildtype gastrulation is observed in late gastrula (48hpf) stage embryos treated with standard control morpholino or dextran as assessed by fluorescent phalloidin labeling and In-situ hybridization of molecular markers for oral (*NvWntA, NvWnt1*, *NvWnt2*, *NvWnt3*, *NvWnt4*, *NvFoxA*, *NvBrachyury*, and *NvSnailA*) and aboral (*NvSix3/6*, *NvFgf2A*, *NvSfrp1/5*, and *Dkk1/2/4*) patterning. Control treatments produced no observable changes in *NvAx6* or *NvAx1* expression assessed at early gastrula (24hpf) stages. Images were compiled from at least three separate experiments and the number of similar phenotypes is noted as a fraction in the lower left hand corner.

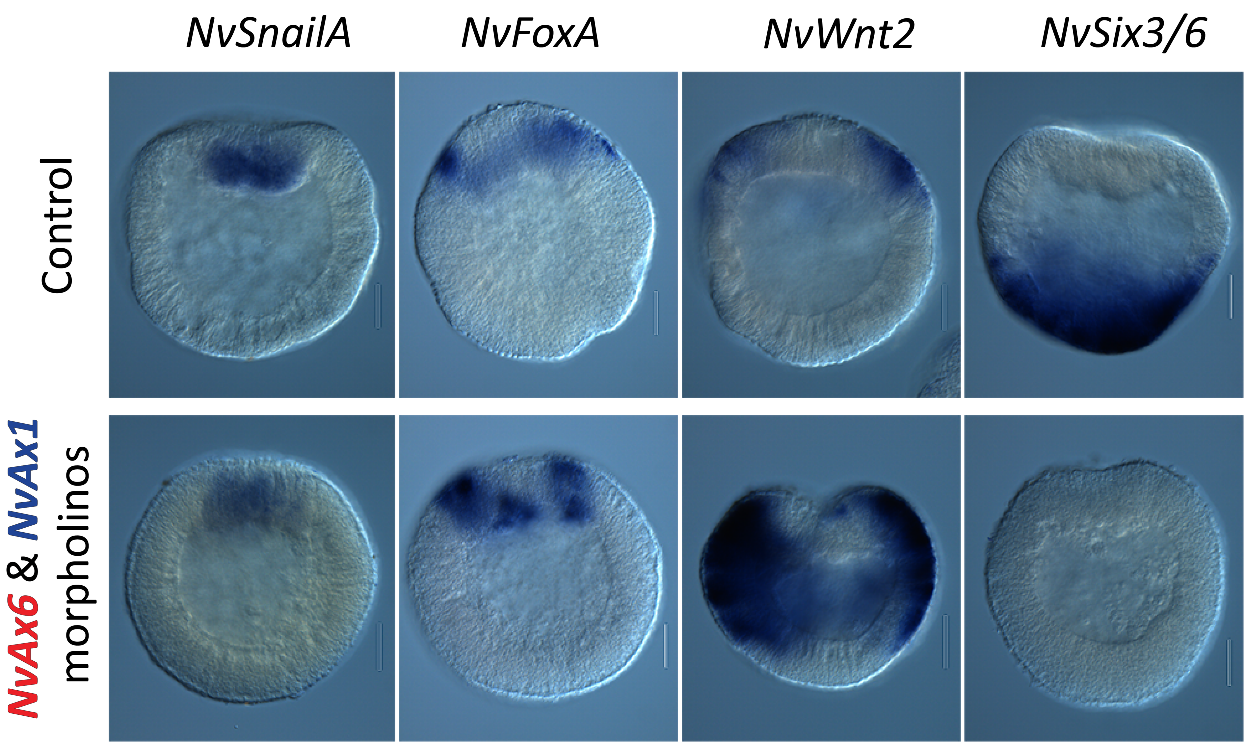

Supplementary Figure 3 Disruption of both oral and aboral *Hox* genes abolishes aboral expression prior to gastrulation

Defects resulting from co-injection experiments as assessed by *in situ* hybridization for oral (*NvSnailA and NvFoxA*), oral-aboral boundary (*NvWnt2*), and aboral (*NvSix3/6*) markers at early gastrula stages (24hpf). Images were compiled from at least three separate experiments and the number of similar phenotypes is noted as a fraction in the lower left hand corner.

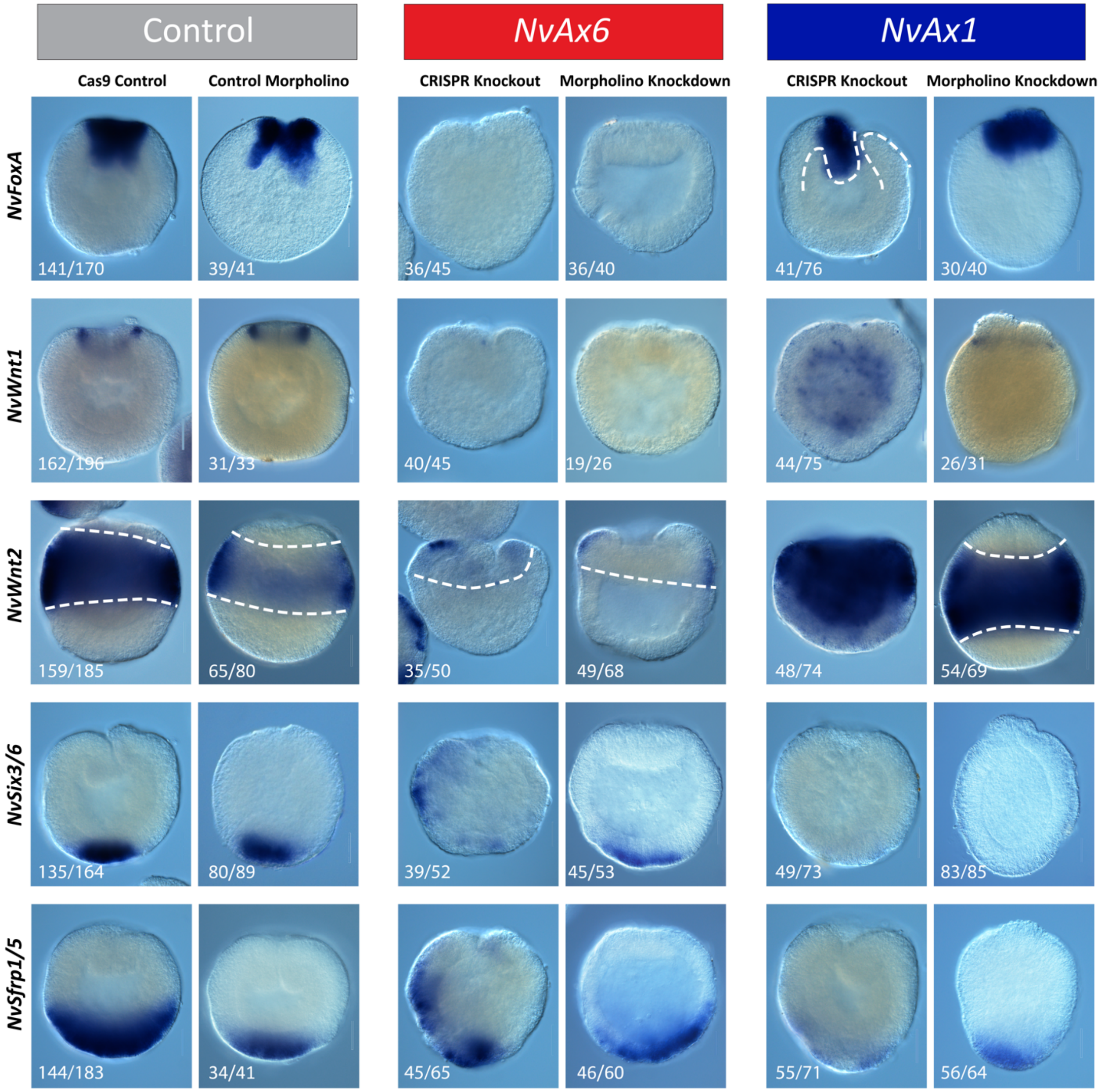

Supplementary Figure 4 Results of CRISPR Cas9 mediated knockout of anterior and central/posterior *Hox* genes on gastrula stage axial patterning

In situ hybridization of select molecular markers for oral-aboral axis specification at late gastrula (48hpf) stages in Cas9 and morpholino control treatments **(grey),** morpholino knockdown and CRISPR/Cas9 mediated knockout of the anterior *Hox* gene (*NvAx6*) **(red)**, and morpholino knockdown and CRISPR/Cas9 mediated knockout of the central/posterior *Hox* gene (*NvAx1*) **(blue)**. White dashed lines in *NvFoxA* panels are used to highlight the pharynx. White dashed lines in *NvWnt2* panels serve to highlight changes in the expression domain. Images were compiled from at least three separate experiments and the number of similar phenotypes is noted as a fraction in the lower left hand corner.

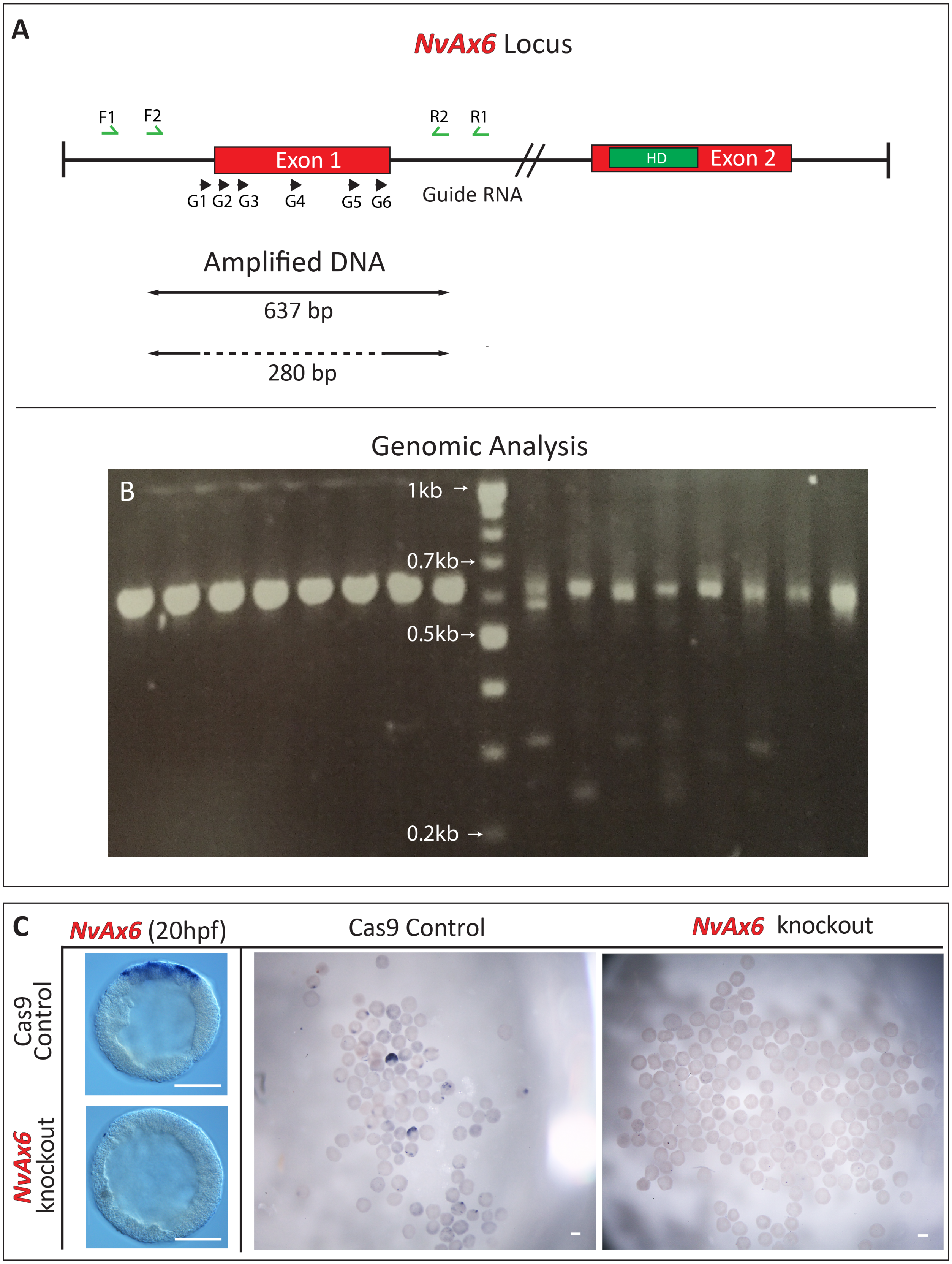
A
NvAx6 Locus
F1
F2
R2
R1
Exon 1
HD
Exon 2
G1 G2 G3
G4
G5 G6
Guide RNA
Amplified DNA
637 bp
280 bp
Genomic Analysis
B
1kb →
0.7kb→
0.5kb→
0.2kb →
C
NvAx6 (20hpf)
Cas9 Control
NvAx6 knockout
Cas9 Control
NvAx6 knockout

Supplementary Figure 5 Cas9 mediated excision of the *NvAx6* locus

(a) Diagram of the *NvAx6* locus. Line represents topography of genomic DNA. Red boxes represent the two exons of the *NvAx6* locus. The Green box represents approximate location and size of the Homeobox. Black arrows show approximate location and orientation of guide RNA (G1-G6) binding sites (Supplementary table 1). Green arrows show approximate location and orientation of primary (F1,R1) and secondary (F2,R2) primer sets used to amplify a 637bp region of the *NvAx6* locus using nested PCR (primers listed in Supplementary table 1). The amplified region of DNA would be reduced to 280bp in an excision involving G1 and G6. **(b)** Amplified section of genomic DNA isolated from single early gastrula (24hpf) stage embyros treated with cas9 protein (left of DNA ladder) and with cas9 protein and guide RNAs G1-G6 (right of the ladder). **(c)** In-situ hybridization shows the loss of *NvAx6* expression at late blastula (18hpf) stages. Images were compiled from at least three separate experiments and the number of similar phenotypes is noted as a fraction in the lower left hand corner. Scale Bars are 100 µm.

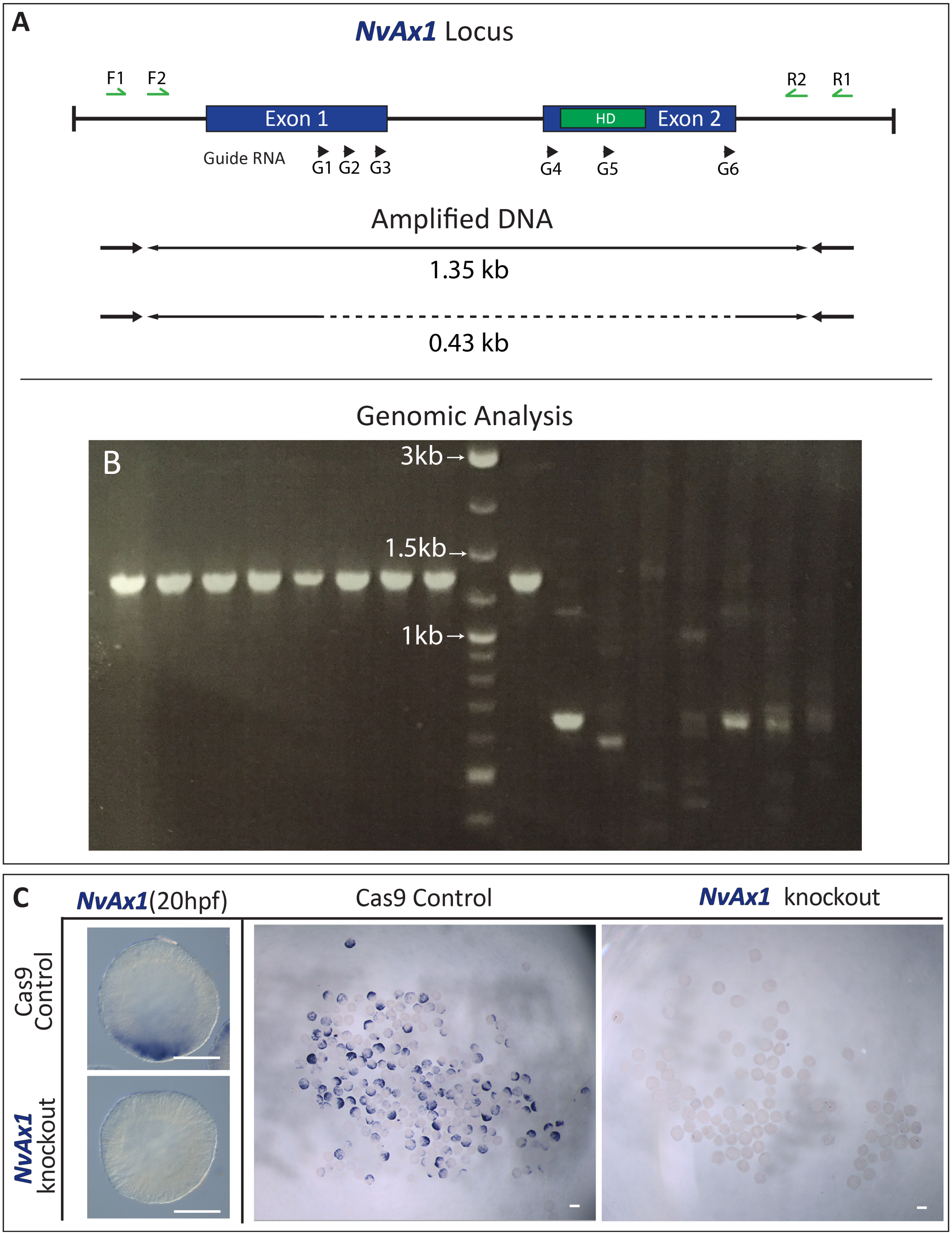
A
NvAx1 Locus
F1
F2
R2
R1
Exon 1
HD
Exon 2
Guide RNA
G1 G2 G3
G4
G5
G6
Amplified DNA
1.35 kb
0.43 kb
Genomic Analysis
B
3kb→
1.5kb→
1kb→
C
NvAx1(20hpf)
Cas9 Control
NvAx1 knockout
Cas9 Control
NvAx1 knockout

Supplementary Figure 6 Cas9 mediated excision of the *NvAx1* locus
(a) Diagram of the *NvAx1* locus. Line represents topography of genomic DNA. Blue boxes represent the two exons of the *NvAx1* locus. The Green box represents approximate location and size of the Homeobox. Black arrows show approximate location and orientation of guide RNA (G1-G6) binding sites (Supplementary Table 1). Green arrows show approximate location and orientation of primary (F1,R1) and secondary (F2,R2) primer sets used to amplify a 1.35kb region of the *NvAx1* locus using nested PCR (primers listed in Supplementary table 1). The amplified region of DNA would be reduced to 0.43kb in an excision involving G1 and G6. **(b)** Amplified section of genomic DNA isolated from single early gastrula (24hpf) stage embryos treated with cas9 protein (left of DNA ladder) and with cas9 protein and guide RNAs G1-G6 (right of the ladder). **(c)** In-situ hybridization shows the loss of *NvAx1* expression at late blastula (18hpf) stages. Images were compiled from at least three separate experiments and the number of similar phenotypes is noted as a fraction in the lower left hand corner. Scale Bars are 100 µm.

Supplementary Table 1 Primer sequences for CRISPR/Cas9 knockouts

Oligo Sequences for gRNAs targeting the *NvAx6 Locus*

Name	Target Sequence (5'-3')	Oligo Sequence (5'-3')	Efficiency Score
G1_Ax6	GACACATTTGGGCATGAG	AATATTTAGGTGACACTATAGACACATTTGGGCATGAGGTTTTAGAGCTAGAAATAGC	5.27578
G2_Ax6	GGTGGCGAGTGGCGCTGT	AATTAATACGACTCACTATAGGTGGCGAGTGGCGCTGTGTTTTAGAGCTAGAAATAGC	6.64511
G3_Ax6	GGATGTCCTTACCGCATTT	AATTAATACGACTCACTATAGGATGTCCTTACCGCATTTGTTTTAGAGCTAGAAATAGC	3.66894
G4_Ax6	GGCTCAGGAATTCCGAGACA	AATTAATACGACTCACTATAGGCTCAGGAATTCCGAGACAGTTTTAGAGCTAGAAATAGC	4.49472
G5_Ax6	GGCAAACGGCCGACTTTCGA	AATTAATACGACTCACTATAGGCAAACGGCCGACTTTCGAGTTTTAGAGCTAGAAATAGC	5.88989
G6_Ax6	GGATATTCACCAAAAGAAA	AATTAATACGACTCACTATAGGATATTCACCAAAAGAAAGTTTTAGAGCTAGAAATAGC	5.27578

Oligo Sequences for gRNAs targeting the *NvAx1* locus

Name	Target Sequence (5'-3')	Oligo Sequence (5'-3')	Efficiency Score
G1_Ax1	GAGCCTATGCAGTCATTCCA	AATATTTAGGTGACACTATAGAGCCTATGCAGTCATTCCAGTTTTAGAGCTAGAAATAGC	6.48921
G2_Ax1	GATCACCAGAGCTACTTCTC	AATATTTAGGTGACACTATAGATCACCAGAGCTACTTCTCGTTTTAGAGCTAGAAATAGC	5.5148
G3_Ax1	GGTGGCCGAACTCCACGAC	AATTAATACGACTCACTATAGGTGGCCGAACTCCACGACGTTTTAGAGCTAGAAATAGC	6.29104
G4_Ax1	GGCTGGAGTAATCGAGCTGA	AATTAATACGACTCACTATAGGCTGGAGTAATCGAGCTGAGTTTTAGAGCTAGAAATAGC	4.75834
G5_Ax1	GGCTTCGCAGTTAAACCTCA	AATTAATACGACTCACTATAGGCTTCGCAGTTAAACCTCAGTTTTAGAGCTAGAAATAGC	4.29472
G6_Ax1	GAACTGTATGGGCTTCAG	AATATTTAGGTGACACTATAGAACTGTATGGGCTTCAGGTTTTAGAGCTAGAAATAGC	4.5671

trans-activating crRNA (tracrRNA) (5'-3')
GATCCGCACCGACTCGGTGCCACTTTTTCAAGTTGATAACGGACTAGCCTTATTTTAACTTGCTATTTCTAGCTCTAAAAC

Forward and reverse primers for amplifying targeted regions the *NvAx6* and *NvAx1* loci via nested PCR

NvAx6 (5'-3')	
F1	GGGGCGTCAGTTGCAAATTT
F2	GTTGCAGACCGGGACAGTAA
R1	AGACTAGTACCAAGACAGGCGA
R2	GTGTGCGTGTTTGGCTTTCT

NvAx1 (5'-3')	
F1	TCTAGGGCGGTTTTAAGGCG
F2	CGACAAGTGTTCGTTTCGGT
R1	TCTCCAGGAGCAGGGAAAGA
R2	TGCGTATCTACATTTTGTCTTGGAA

NvAnthox6
ATGAGCGGCGGTGGCGAGTGGCGCTGTAGGATGTCCTTACCGCATTTAGGCATGGCCTCAAACTTGCAGCCACAACGCTACA
GTAACAATTTGGACTTTGGGCAAACAGTCCCCCTTACGCACGCACCAGTTCCAACAAGCCCTGTTGTGGGCTCAGGAATTCCGA
GACAAGGACTCTACTACCATTCATCCATCTCTGCTGCGCAGAAACCATCGACACAGCCTGAACAACAAATCTTCCAACACCAAA
CTTCCACCAAATGTCAGGCGCAAAAGAAAAACGCTCAGGCGGAGAAGAAGCGTTGGCAAACGGCCGACTTTCGATGGATATTC
ACCAAAAGAAAAGCTACAGGGCCAGCGTCCATCAACACGAAAATTCCTGAAGAGAAAGAGCAAAACCCTTCACCGAGTCAGAA
GAAACGATTCACATTCACACAAAGGCAACTTGTCGAGCTAGAAAAAGAATTCCATTTCAGCAAATATCTCACGAGAACACGGCG
AATCGAGATTGCTACAACTCTAAAACTCACTGAAATGCAAATCAAAATCTGGTTTCAAAATCGAAGAATGAAATGGAAAAGGGAG
TTTAAGGATTCCTTGCAAAAACCAGCGAACGGCGAGCCTACGTACCAAAACTCAAGCTTTGTTCAGCCTTTCCACAATGCACCC
TTCAACGGCAACCATTCAACTACGATGGCATTAAACGGACTTGCGTACTATCCAAGCTACATGTCTGGGTTTGGGTAA

NvAnthox6a
ATGAGTGTACAGAGGGGAGAGAACCGCTCGAATCAGAGCCCATCCTTGACCCTAGAGGCTGAGTCCCGAAACACGAGCGCGC
AACGCAGTCCTCAAGCTGAAAACACCGCTACGAATGGAGAATCCCAGAGGAAACAAATCTACCCCTGGATGACTGAATTCCGG
GTTAAAGGTCCCCCGCCACAAACCAAGGAGTGCACATCCGATAAAAACCGGACGATCTACTCCACTCGACAATTGGTAGAGCT
GGAAAAAGAATTCCACTACAATCGCTACCTCTGCCGACCTAGGAGGATCGAAATCGCCCAGTCTTTAGAACTCACTGAAAAGCA
GGTTAAAATCTGGTTTCAGAATAGAAGAATGAAATGGAAAAAAGAAAACAAACTTGTAGAAAAAGCAATCGAGAGTGAAATCAAG
GAAAGAGTAAAGAATTTGAGCACACAGAACCCAGATGGGTTTGGCAGCTTACCTATGTACGCGAACGATTTGAACTCACTTCAA
CAAATGTACGATTTGAGCAGCTCCTGTACTGCCTTCCAGCCTGTTAGCGCCTTTGCTAGCGGCTTTGGAGGCAGTTCTATGCAC
AATGGTGGACAAAGATCAGTGTGGCCTCACTTCAGCTGA

NvAnthox7
ATGAACTACTCTGCGCTTTTTCCAAGCTTTGACACTTCTGTCACTTGGGACCCGTCACGTCATCACCTCAAAAACACAGAAAATC
CCGGCACCTTGCCCGAAACTCAAGGGTTTGGTGCTGGCCTTTTTCTTGACACTTACACGAGCCATATAGGAAGGGACTTTGATG
GGCCCATAGGTAGCATACCGGTTGGAAGCTCCAATCGTGAATCACTCCCAAACTATCTTCTACAGCCCATCTACCCATGGATGA
GGACAAAAAAATCCGGCTCTCGCGGAATAGGCGGCAAGCACACCAAGAGGTACCGCACGAGCTACACAAACCGACAACTTCTT
GAGCTCGAGAAAGAGTTTCACTACAATAAATACTTATGCGGCACTCGAAGGCGAGAGCTGGCCAACGCTATGAAGCTCACAGA
GAGACAAGTCAAAGTTTGGTTCCAAAACAGGCGCATGAAACTCAAGAAGGACGAGAAGCAGAAAGAAGACGGACCAAGCGCCT
TCCCGGATTATGATGTGATGAAGTCTCTTCCTCGTCTTCCAAGGCTACTGCCAACATACGCAACTCAAACGGAAGGCTACCCTC
CAATAGAGGCCGTAGAGCACCTCGACAGTCAGTCACAAAACGCACTAAGACATTACACCTATAGCAATTACTCCATGCTAACGC
ACGCGCTCAATACAGTCCTGCAGTGA

NvAnthox8
ATGGACATGTCAGGCCCCTATAAATCACCAGTGATAAACAAAACTTATCCGAGCTCGGAAACCATGCTCTGGTCCAGCCCGCCA
AGACTCGCACATCTGAATAGTTGCCTCTACACACAACCCAGGAAATCTCTATACACGTACGGAGAAGGCGAGAGGGCTAACGA
GATCCTACAGGGGGAGCACTCTAGTAGACCGAACTACAGTTACCCTGTCCCGAATGAGAATCAAGGAAGCCAGAGTGAGCCTA
TCACCCAGCATCTTCTCGATCCTATCTACCCATGGATGAAGCCCAAGAAGGGAGGCAAAAATCACTTTGCTTTTGTTAGAGAATC
AAAGAGGCACCGAACGAGTTACACCAACAAACAGCTCCTGGAGCTCGAGAAAGAATTCCACTTCAACAAATACCTCTGTAGCTC
ACGCCGAAGAGAAATCTCCAAAGCCCTGCAGTTGACGGAGCGTCAAGTGAAAATTTGGTTTCAGAACCGCCGAATGAAATGGA
AAAAAGACGAGAAACAGAAAACAGAGGACTGTTATAGTGCGCATCAAGAGAACATCGAGGAAATTCCATTATCGTCGGCACTAG
CGCCATTGTACATTCCAAACTCTCGGTCAGCGTGCCTTCACTCCTGA

NvAnthox1a
ATGGATTGCTACTTCCCGGTAACTAACACTTTCAATCCGCCTGCGTTTTGCGCAATGCCGTCTATGATGTACACTTACGGAATGC
GGTCATACTATCCGCCTGCCGCATTGTACCCACTAGCTAGCGCCTCCCTAACTGAGAGGTCAAACGGACCCATAATGAACTCTC
AAGTACAGGCAGATCAGAACTCGGCAATAAATCAAGGCATGGAGCTAAGAAACACAGCAAGTAGCGGCAAGCTTCAGATGAGT
GGCAAGGGCTGTGATAGCTATGTGAGGCTTCTCTCAAGGAATGAACCCGCTGAAAAAGAACTCGAGAGGTCTGACGACGAATG
TGAAGATTCTAAAAGTGAGGAGAGTGGCGACGCTCCGTGGCACTACAAGCAACCATTCAAACACAGGAAGCGAATGGCATACA
CCCGCATACAACTCCTAGAGCTGGAAAAAGAGTTCCATTTTACGAGATATTTGACAAAAGAGCGACGCACAGAGATGGCTAGAA
TGCTTGATCTAACAGAACGTCAAGTAAAAATCTGGTTTCAAAACCGTAGGATGAAATGGAAGAAGGACAATAAACCAGTTATTCC
TACGAGTCGAGGAATGCGCAAGGGCTGCACGCGTTAG

NvAnthox1
ATGCAGTCAACTCAAACATACCCGGCGTATTACGGCACGGCTACCGAAAAACCTCAATCGCATGTTCAGTCACTGGACTTTCCA
TACTCTCAATGCCGAATGCAATCGCCCAACTACTACGGGCAAACTTCTATGCCGAATTTTATGGCCGCTATGCCAAGCAACACT
CATTCGACCCAAGTCAACGGCTATAGCCCCGAGCCTATGCAGTCATTCCAGGGACAAATGCCGAATACAAACAATACAGCAAGT
CCAATAGATCACCAGAGCTACTTCTCAGGTAGCGCTGAACCAGGGCAGAACTGTTCGTTCCATCAGTACGCTTGGCTTAAAAGT
ACCGCTCCTGATAACTGGTGGCCGAACTCCACGACAGGGAACTCCGGCTGGAGTAATCGAGCTGAGGGCAAGCGCAAGAGGA
CTGCGTATACGAGAAAGCAACTTCTGGAGTTAGAGAAAGAATTCCACTTTAATCACTTCTTAACAAAAGAGCGGAGATCTGAAAT
GGCTTCGCAGTTAAACCTCACGGAAAGGCAAGTTAAAATCTGGTTCCAAAATAGGCGGATGAAATGGAAAAAATGCAATGCGCA
AGCCCTGACAAAAACCTCCAAGTCACCATCGCAGGGTGGCCTTTCAACATCGGGAAGACAGGACTTACATAATATGAACAACTC
CACGACGCCTTCACAGCACAACTCAGAACAACTAACTACTACGAATCAAATCTCCAACTCTCAATTGCAGAACTGTATGGGCTTC
AGGGGGCATTAG

Supplementary references

1. Pascual-Anaya, J., D'Aniello, S., Kuratani, S. & Garcia-Fernàndez, J. Evolution of Hox gene clusters in deuterostomes. *BMC Dev. Biol.* **13,** 26 (2013).
2. DuBuc, T. Q., Ryan, J. F., Shinzato, C., Satoh, N. & Martindale, M. Q. Coral comparative genomics reveal expanded Hox cluster in the cnidarian-bilaterian ancestor. *Integr. Comp. Biol.* **52,** 835–41 (2012).
3. Chourrout, D. *et al.* Minimal ProtoHox cluster inferred from bilaterian and cnidarian Hox complements. *Nature* **442,** 684–687 (2006).
4. Ryan, J. F. *et al.* Pre-bilaterian origins of the hox cluster and the hox code: Evidence from the sea anemone, Nematostella vectensis. *PLoS One* **2,** e153 (2007).
5. Baumgarten, S. *et al.* The genome of Aiptasia , a sea anemone model for coral symbiosis. *Proc. Natl. Acad. Sci.* **112,** 11893–11898 (2015).
